# Brain insulin resistance and Alzheimer’s disease: a systematic review

**DOI:** 10.1590/1980-5764-DN-2023-0032

**Published:** 2024-02-09

**Authors:** Luis Jesuino de Oliveira Andrade, Luís Matos de Oliveira, Alcina Maria Vinhaes Bittencourt, Letícia Góes de Carvalho Lourenço, Gabriela Correia Matos de Oliveira

**Affiliations:** 1 Universidade do Estado de Santa Cruz, Department of Health, Ilhéus BA, Brazil.; 2 Escola Bahiana de Medicina e Saúde Pública, Salvador BA, Brazil.; 3 Universidade Federal da Bahia, School of Medicine, Salvador BA, Brazil.; 4 Faculdade Pernambucana de Saúde, Recife PE, Brazil.; 5 Programa de Saúde da Família, Salvador BA, Brazil.

**Keywords:** Alzheimer’ Disease, Insulin Resistance, Brain, Cognition, Systematic Review, Doença de Alzheimer, Resistência à Insulina, Encéfalo, Cognição, Revisão Sistemática

## Abstract

The disability of cells to react to insulin, causing glucose intolerance and hyperglycemia, is referred to as insulin resistance. This clinical condition, which has been well-researched in organs such as adipose tissue, muscle, and liver, has been linked to neurodegenerative diseases like Alzheimer’s disease (AD) when it occurs in the brain.

**Objective:**

The authors aimed to gather data from the current literature on brain insulin resistance (BIR) and its likely repercussions on neurodegenerative disorders, more specifically AD, through a systematic review.

**Methods:**

A comprehensive search was conducted in multiple medical databases, including the Cochrane Central Register of Controlled Trials, EMBASE, Medical Literature Analysis and Retrieval System Online (Medline), and PubMed^®^, employing the descriptors: “insulin resistance”, “brain insulin resistance”, “Alzheimer’s disease”, “neurodegeneration”, and “cognition”. The authors focused their search on English-language studies published between 2000 and 2023 that investigated the influence of BIR on neurodegenerative disorders or offered insights into BIR’s underlying mechanisms. Seventeen studies that met the inclusion criteria were selected.

**Results:**

The results indicate that BIR is a phenomenon observed in a variety of neurodegenerative disorders, including AD. Studies suggest that impaired glucose utilization and uptake, reduced adenosine triphosphate (ATP) production, and synaptic plasticity changes caused by BIR are linked to cognitive problems. However, conflicting results were observed regarding the association between AD and BIR, with some studies suggesting no association.

**Conclusion:**

Based on the evaluated studies, it can be concluded that the association between AD and BIR remains inconclusive, and additional research is needed to elucidate this relationship.

## RESUMO.

A incapacidade das células de reagir à insulina, ocasionando intolerância à glicose e hiperglicemia, é chamada de resistência à insulina. Essa condição clínica, que tem sido bem pesquisada em órgãos como tecido adiposo, músculo e fígado, tem sido associada às doenças neurodegenerativas como a doença de Alzheimer (DA) quando ocorre no cérebro.

**Objetivo:**

O objetivo dos autores foi reunir os dados da literatura atual sobre a resistência insulínica cerebral (RIC) e sua provável repercussão em doenças neurodegenerativas, mais especificamente na DA, por meio de uma revisão sistemática da literatura.

**Métodos:**

Foi realizada uma pesquisa abrangente em vários bancos de dados médicos, incluindo o Cochrane Central Register of Controlled Trials, EMBASE, Medical Literature Analysis and Retrieval System Online (Medline) e PubMed, empregando os descritores: “resistência à insulina”, “resistência insulínica cerebral”, “doença de Alzheimer”, “neurodegeneração” e “cognição”. Os autores concentraram sua busca em estudos no idioma inglês publicados entre 2000 e 2023 que investigaram a influência da RIC em distúrbios neurodegenerativos ou ofereceram *insights* sobre os mecanismos subjacentes da RIC. Dezessete estudos que atenderam aos critérios de inclusão foram selecionados.

**Resultados:**

Os resultados demonstram que a RIC é um fenômeno observado em uma variedade de doenças neurodegenerativas, incluindo a DA. Estudos sugerem que a utilização e captação prejudicadas de glicose, a produção reduzida de trifosfato de adenosina (ATP) e as alterações na plasticidade sinápticas causadas pela RIC estão ligadas a problemas cognitivos. No entanto, foram observados resultados conflitantes com relação à associação entre DA e RIC, com alguns estudos sugerindo nenhuma associação.

**Conclusão:**

Com base nos estudos avaliados, pode-se concluir que a associação entre DA e RIC ainda é inconclusiva, e pesquisas adicionais são necessárias para elucidar essa relação.

## INTRODUCTION

Alzheimer’s disease (AD) is a gradually progressive type of neurodegenerative disease characterized by the loss of memory and cognitive abilities, and challenges with daily activities^1^. It is the most frequent form of dementia, responsible for over 60% of all cases, affecting approximately 47 million individuals globally, with estimates that this number will triple by 2050^2^. The underlying pathophysiology of AD remains complex and is not fully understood. Still, a growing body of studies indicates that brain insulin resistance (BIR) may perform a significant role in its onset and progression^3^.

Insulin is a fundamental hormone in controlling glucose metabolism and corporal energetic homeostasis. It performs an important function in signaling pathways that command cell growth, survival, and neuronal activity in the brain^4^. Insulin resistance (IR) is a status in which the body’s tissues, including the brain, exhibit a low insulin response, leading to high levels of insulin in the blood^5^. If left untreated, IR can progress to type 2 diabetes (DM2), which is a well-established risk factor for AD^6^.

In the last few years, increasing interest has occurred in the relationship between BIR and AD. Several observational studies reported that individuals with DM2 or IR have a higher threat of developing AD^7^. These studies also suggested that BIR highly favors the pathogenesis of AD through a variety of systems, such as amyloid-beta accumulation, tau protein hyperphosphorylation, inflammation, oxidative stress, and neurovascular dysfunction^8^.

Despite the increased interest in this field of knowledge, there is currently no consensus on the relationship between BIR and AD. Previous systematic reviews and meta-analyses presented conflicting results, and the quality of the evidence is limited by several factors, including small sample sizes, inconsistent definitions of BIR, and variations in study design and population characteristics^8^ ^,^ ^9^. Furthermore, recent studies identified novel molecular and cellular mechanisms that may be behind the link between BIR and AD, highlighting the need for an updated and comprehensive review of the existing literature^10^.

Therefore, this systematic review aimed to provide a comprehensive summary of the current evidence on the relationship between BIR and AD. Specifically, the present study systematically reviewed the evidence for BIR in AD, including its prevalence, temporal relationship, and underlying pathophysiological mechanisms; analyzed the relationship between BIR and cognitive impairment, dementia severity, and other clinical outcomes in AD; and identified key research gaps and future directions for investigating the role of BIR in the pathogenesis and treatment of AD.

## METHODS

### Search strategy

Preferred Reporting Items for Systematic Reviews and Meta-Analyses (PRISMA) is a standardized approach to the development and reporting of systematic reviews and meta-analyses^11^. In the case of this systematic review of BIR and AD, PRISMA was a tool for ensuring that this review was conducted in a rigorous, methodical, and transparent manner.

A comprehensive search of numerous electronic databases (PubMed, Medline, EMBASE, and the Cochrane Central Register of Controlled Trials) was performed for articles edited between 2000 and 2023, using the keywords “insulin resistance”, “brain insulin resistance”, “Alzheimer’s disease”, “neurodegeneration”, and “cognition”. The search was complemented by a manual evaluation of the reference lists of relevant papers.

### Study selection

Studies that were incorporated into the research obeyed certain criteria such as:

- Human studies investigating the association between BIR and AD;
- Observational studies (cross-sectional, case-control, cohort) and randomized controlled trials (RCTs);
- Studies reporting data on measures of IR or biomarkers of insulin signaling;
- Studies reporting data on clinical outcomes related to AD; and
- Studies published in the English language.

### Data extraction

Data were obtained from selected studies using a standardized form. Data extracted and included in the study design were participant characteristics, measures of IR, clinical outcomes, and effect size estimates. The study level of qualification was evaluated through the Cochrane risk of bias tool for RCTs, and the Newcastle-Ottawa importance valuation grade for observational studies.

### Data analysis

A systematic review of BIR and AD involved synthesizing and analyzing data from multiple studies to determine the relationship between these two conditions. The process comprised the following steps:

- Formulating a research question and search strategy. The first step was to define and identify the key terms and databases to be searched. In this case, the research question was: “What is the relationship between BIR and the risk of AD?” The search strategy included keywords such as “insulin resistance” and “Alzheimer’s disease”.
- Screening and selecting studies. The next step was to screen the resulting studies for relevance and quality, which involved several rounds of screening. The first round was based on the abstracts and titles of the studies and subsequent rounds were based on full-text articles. Only studies that met both the inclusion criteria and a predefined level of quality were eligible: searches from the previous ten years, studies published in English, with patients diagnosed with AD using established clinical criteria. Exclusion criteria: animal model publications, review articles, and conferences.
- Extracting data and assessing bias. Once the studies were selected, the next step was to extract relevant data from each study. The studies were also assessed for bias, considering factors such as study design, sample size, and potential sources of confounding.
- Synthesizing data and calculating effect sizes. The extracted data were combined and analyzed using statistical methods to calculate effect sizes that represent the magnitude and direction of the association between BIR and AD. These effect sizes were grouped and analyzed using meta-analysis techniques to estimate the overall effect size across all included studies.
- Interpreting results and concluding. The results of the systematic review were interpreted and conclusions were drawn.
- The risk of bias was assessed for each included study using the Quality Assessment of Diagnostic Accuracy Studies (QUADAS)-2 risk of bias assessment tool.

## RESULTS

**Figure 1.**
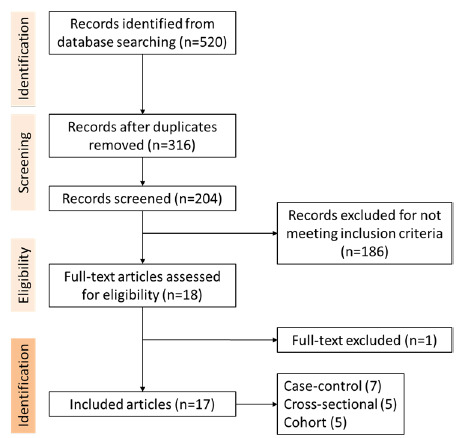
Flowchart on the selection process of eligible studies.

The results of our search strategy are summarized in a PRISMA flow diagram (Figure 1) and demonstrate the stages of obtaining studies for inclusion in the review. A total of 520 papers were identified through electronic database search using pre-defined search terms and were subsequently examined by title and abstract. After removing duplicates, 204 articles underwent screening of abstracts and full-text review, and were selected if they reported the association between BIR and AD. Finally, 17 works fulfilled the inclusion criteria and were selected for data extraction and analysis.

All the selected articles analyzed the link between BIR and AD. Of the 17 works that met the inclusion criteria, seven were case-control studies, five were cross-sectional, and five were cohort studies. The surveys were performed in different countries, and sample sizes ranged from 18 to 5,586,048 participants.

### Characteristics of the included studies

- Reference: Su et al.^12^; Type of study: Case-control study; Objective: “Longitudinal study for an average of 35 months to assess cognitive decline over time”; Participants: 87 mild cognitive impairment subjects and 135 matched controls; Diagnosis method of AD: National Institute on Aging-Alzheimer’s Association workgroups on diagnostic guidelines for Alzheimer’s disease; Diagnosis criteria of BIR: Genes that have been demonstrated to mediate IR in AD brains; Covariates studied: Age, gender, education, diabetes. Conclusion: “The results suggested that the restoration of insulin activity represents a promising therapeutic target for improve cognitive decline in AD”.
- Reference: Niyasti et al.^13^; Type of study: Case-control study; Objective: “Polymerase chain reaction was performed to amplify a DNA segment of 263 base-pair length containing the single nucleotide polymorphism”; Participants: 150 patients with late-onset AD and 150 unrelated healthy controls; Diagnosis method of AD: National Institute on Aging and Alzheimer’s Association; Diagnosis criteria of BIR: Rs1801278 polymorphism in the insulin receptor substrate-1; Covariates studied: Age, sex, genotype. Conclusion: “Association of Insulin Receptor Substrate-1 Gene Polymorphism (rs1801278) present at the BIR is significantly associated with the risk of developing AD”.
- Reference: van der Velpen et al.^14^; Type of study: Case-control study; Objective: “Paired plasma and cerebrospinal fluid samples” (CSF); Participants: AD group (n=40) and cognitively healthy controls without cerebral AD pathology (n=34); Diagnosis method of AD: Mini-Mental State Examination (MMSE), Clinical Dementia Rate, quotient albumin or plasma/albumin ratio; Diagnosis criteria of BIR: Metabolic profiling untargeted metabolomics (tricarboxylic acid cycle, and its anaplerotic pathways, as well as the neuroactive tryptophan and kynurenine pathway) and targeted quantification (identified deregulated pathways highlighted); Covariates studied: Age, sex, body mass index (BMI), cognitive function, CSF biomarkers, biochemical measures. Conclusion: “The study showed the translational potential of the pathway quantitative to assess central nervous system metabolic defects which are part of the AD pathophysiology”.
- Reference: Andalib et al.^15^; Type of study: Case-control study; Objective: “To test the hypothesis of association of late onset Alzheimer’s disease (AD) with DM2 in an Iranian population”; Participants: 243 subjects including 81 patients with late onset AD and 162 healthy controls; Diagnosis method of AD: Clinical examination, neuropsychiatric tests, and magnetic resonance imaging criteria of the National Institute on Aging-Alzheimer’s Association workgroups; Diagnosis criteria of BIR: Physician medical record diagnosing DM2, or use of oral hypoglycemic agents; Covariates studied: DM2, age, gender. Conclusion: “The findings of study showed that IR and DM2 is associated with higher risk of AD”.
- Reference: Mullins et al.^16^; Type of study: Case-control study; Objective: “Use Magnetic Resonance Spectroscopy to assess AD-related differences in the posterior cingulate/precuneal ratio of glucose, lactate, and other metabolites”; Participants: 27 older controls, and 27 younger control participants; Diagnosis method of AD: MMSE, clinical dementia rating, CSF Aβ 1-42, CSF B-amyloid 42, p-Tau181P, Tau phosphorylated on threonine 181; Diagnosis criteria of BIR: Homeostasis Model Assessment (HOMA) 2 beta cell function; HOMA2%B, HOMA2%S, HOMA 2 insulin sensitivity; HOMA2 IR, HOMA 2 IR; Covariates studied: DM2, age, sex, glucose, lactate and ascorbate; Conclusion: “The study showed substantial elevations in glucose, lactate, and ascorbate levels within the posterior cingulate/precuneus of AD participants”.
- Reference: Xu et al.^17^; Type of study: Case-control study; Objective: “To elucidate the processes that cause neurodegeneration in AD by measuring levels of metabolites and metals in brain regions that undergo different degrees of damage”; Participants: nine AD patients and nine controls; Diagnosis method of AD: Employed mass spectrometry to measure metabolites and metals in brain regions; Diagnosis criteria of BIR: Measure glucose levels, fructose levels, and sorbitol levels in brain regions; Covariates studied: Age, sex, brain-weights, and post-mortem delay; Conclusion: “Elevation of brain glucose and deficient brain copper potentially contribute to the pathogenesis of neurodegeneration in AD”.
- Reference: Tortelli et al.^18^; Type of study: Cohort study; Objective: “To evaluate midlife metabolic profile and the risk of late-life cognitive decline”; Participants: 797 subjects; Diagnosis method of AD: Multicenter Italian Study on Cholelithiasis (MICOL Study) and MMSE; Diagnosis criteria of BIR: HOMA index; Covariates studied: Age, gender, education level (years), BMI, systolic and diastolic blood pressure; Conclusion: “The control blood glucose levels, regardless of a diagnosis of diabetes mellitus, as early as midlife prevents late-life dementia”.
- Reference: Abner et al.^19^; Type of study: Cohort study; Objective: “SMART database that comprises a standardized set of data elements contributed by 11 longitudinal studies of aging and cognition”; Participants: 2,365 autopsies; Diagnosis method of AD: Alzheimer’s neuropathological changes; Diagnosis criteria of BIR: Known diabetes status; Covariates studied: Age at death, gender, Apolipoprotein E (APOE)-ε4; Conclusion: “The study concluded that diabetes increases the risk of cerebrovascular but not AD”.
- Reference: Chung et al.^20^; Type of study: Case-control study; Objective: “Investigate whether genome-wide significant loci of type 2 diabetes mellitus are associated with the risk of AD”; Participants: 400 AD patients, and 500 unrelated controls; Diagnosis method of AD: Use of National Institute of Neurological and Communicative Disorders and Stroke and the Alzheimer’s Disease and Related Disorders Association criteria for probable AD; Diagnosis criteria of BIR: 32 genetic variants of 11 genes (CDC123, CDKAL1, CDKN2B, FTO, GLIS3, HHEX, IGF2BP2, KCNJ11, KCNQ1, SLC30A8, and TCF7L2) were selected; Covariates studied: Age, sex, APOE genotypes; Conclusion: “The results suggest that genome-wide significant loci of type diabetes (insulin resistance) play no major role in the risk and cognitive impairment of AD”.
- Reference: Schrijvers et al.^21^; Type of study: Cohort study; Objective: “Investigate whether fasting glucose and insulin levels and IR are associated with the risk of AD and whether this risk is constant over time”; Participants: 3,139 participants of the Rotterdam Study; Diagnosis method of AD: MMSE, Geriatric Mental State schedule, and Cambridge Examination for Mental Disorders of the Elderly; Diagnosis criteria of BIR: Glucose and insulin assessments, HOMA; Covariates studied: Age, sex, educational level, APOE genotype, APOE ε4; Conclusion: “The study suggests that insulin metabolism influences the clinical manifestation of AD only within 3 years”.
- Reference: Willette et al.^22^; Type of study: Cross-sectional study; Objective: “Assess whether the IR is associated with amyloid binding in three AD-sensitive brain areas”; Participants: 186 middle-aged adults; Diagnosis method of AD: MMSE; Diagnosis criteria of BIR: HOMA-IR; Covariates studied: Age, gender, diastolic and systolic blood pressure, magnetic resonance imaging, BMI; Conclusion: “The study demonstrated that IR may contribute to amyloid deposition in brain regions affected by AD”.
- Reference: Morris et al.^23^; Type of study: Cross-sectional study; Objective: “To compare IR in aging and aging-related neurodegenerative diseases, and to determine the relationship between IR and gray matter volume in each cohort using an unbiased, voxel-based approach”; Participants: 20 AD and 22 Parkinson’s disease; Diagnosis method of AD: Tests from the Uniform Data Set; Diagnosis criteria of BIR: HOMA 2 and intravenous glucose tolerance test; Covariates studied: Age, gender, education, gray matter volume, whole-brain volume; Conclusion: “The study supports a potential relationship between IR and brain structure in both normal aging and diagnosed neurodegenerative disease”.
- Reference: Kapogiannis et al.^24^; Type of study: Cross-sectional study; Objective: “To assess brain IR in AD by level of serine-type 1 insulin receptor substrate (IRS-1) and its state of phosphorylation in neural-derived plasma exosomes”; Participants: 26 patients with amnestic mild cognitive impairment; Diagnosis method of AD: MMSE and cognitive subscale of the AD assessment scale; Diagnosis criteria of BIR: Neural-derived plasma exosomal IRS-1 and P-IRS-1 levels; Covariates studied: Age, sex, glucose tolerance test; Conclusion: “Insulin resistance reflected in R values from IRS-1 is higher for patients with AD, and accurately predicts development of AD up to 10 year prior to clinical onset”.
- Reference: Willette et al.^25^; Type of study: cross-sectional study; Objective: “To determine if IR predicts AD-like global and regional glucose metabolism deficits in late middle-aged participants at risk for AD, and to examine if IR predicts variation in regional glucose metabolism is associated with worse cognitive performance”; Participants: 150 cognitively normal; Diagnosis method of AD: Immediate Memory, Verbal Learning & Memory, Working Memory, and Speed & Flexibility; Diagnosis criteria of BIR: Fluorodeoxyglucose positron emission tomography and Homeostasis Model Assessment of Insulin Resistance (HOMA-IR); Covariates studied: Age, sex, BMI; Conclusion: “The results show that IR is associated with significantly lower regional cerebral glucose metabolism, which in turn may predict worse memory performance”.
- Reference: Johansson et al.^26^; Type of study: Cross-sectional study; Objective: “Assess whether the serum but not cerebrospinal fluid levels of insulin-like growth factor-I (IGF-I) and IGF-binding protein-3 are increased in AD”; Participants: 60 patients under primary evaluation of cognitive impairment (32 AD) and 20 healthy controls; Diagnosis method of AD: Diagnostic and Statistical Manual of Mental Disorders, Fourth Edition (DSM-IV) and MMSE; Diagnosis criteria of BIR: IGF-I, IGF-binding protein-3, and insulin in serum and CSF; Covariates studied: Age, sex, BMI, waist-hip ratio; Conclusion: “Patients with AD as well as other dementias had high levels of IGF-I in serum but not in CSF. In AD patients, the IGF-I system was associated with biomarkers of AD disease status”.
- Reference: Faqih et al.^27^; Type of study: Retrospective cohort study; Objective: “Study the association between AD and IR and the relation between AD and diabetic patients treated with insulin”; Participants: 354 patients; Diagnosis method of AD: Clinical criteria for AD diagnosis; Diagnosis criteria of BIR: triglyceride-glucose index; Covariates studied: Age, sex, BMI, glycated hemoglobin, cholesterol, triglycerides, high- and low-density lipoprotein cholesterol (HDL and LDL); Conclusion: “The results suggest that AD is associated with IR”.
- Reference: Hong et al.^28^; Type of study: Retrospective, observational, and cohort study; Objective: “Study the potential relationships between the triglyceride glucose index and dementia”; Participants: 5,586,048 participants 40 years of age or older; Diagnosis method of AD: Defined according to the International Classification of Diseases ICD-10 diagnosis codes for dementia (F00, G30, F01, F02, F03, G23.1, G31.0, G31.1, G31.82, G31.83, G31.88, and F10.7); Diagnosis criteria of BIR: Triglyceride-glucose index; Covariates studied: Age, sex, BMI, waist circumference, glycated hemoglobin, total cholesterol, HDL and LDL cholesterol, hypertension; Conclusion: “Triglyceride glucose index was associated with an increased risk of dementia, including AD”.

The summary of the included studies and related outcomes is shown in Table 1.

**Table 1.** Summary of the included studies.

Reference	Year	Design	Sample size	Objective	Conclusions	Risk of bias as per QUADAS-2
Su et al.^12^	2016	Case-control	222	“Longitudinal study for an average of 35 months to assess cognitive decline over time”	“The results suggested that the restoration of insulin activity represents a promising therapeutic target for improve cognitive decline in AD”.	Good-quality
Niyasti et al.^13^	2022	Case-control	300	“Polymerase chain reaction was performed to amplify a DNA segment of 263 base-pair length containing the single nucleotide polymorphism”	“Association of Insulin Receptor Substrate-1 Gene Polymorphism (rs1801278) present at the BIR is significantly associated with the risk of developing AD”	Good-quality
van der Velpen et al.^14^	2019	Case-control	74	“Paired plasma and cerebrospinal fluid samples”	“The study showed the translational potential of the pathway quantitative to assess central nervous system metabolic defects which are part of the AD pathophysiology”.	Low-moderate
Andalib et al.^15^	2019	Case-control	243	“To test the hypothesis of association of late onset Alzheimer’s disease (AD) with DM2 in an Iranian population”	“The evidence from the present study suggested that DM2 was associated with AD in an Iranian population”	Good-quality
Mullins et al.^16^	2018	Case-control	54	“Use Magnetic Resonance Spectroscopy to assess AD-related differences in the posterior cingulate/precuneal ratio of glucose, lactate, and other metabolites”	The study showed substantial elevations in glucose, lactate, and ascorbate levels within the posterior cingulate/precuneus of AD participants	Good-quality
Xu et al.^17^	2016	Case-control	18	“To elucidate the processes that cause neurodegeneration in AD by measuring levels of metabolites and metals in brain regions that undergo different degrees of damage”	“Elevation of brain glucose and deficient brain copper potentially contribute to the pathogenesis of neurodegeneration in AD”	Good-quality
Tortelli et al.^18^	2017	Cohort	797	“To evaluate midlife metabolic profile and the risk of late-life cognitive decline”	“The control blood glucose levels, regardless of a diagnosis of diabetes mellitus, as early as midlife prevents late-life dementia”	Good-quality
Abner et al.^19^	2016	Cohort	2,365	“SMART database that comprises a standardized set of data elements contributed by 11 longitudinal studies of aging and cognition”	“The study concluded that diabetes increases the risk of cerebrovascular but not AD”	Low-moderate
Chung et al.^20^	2015	Case-control	900	“Investigate whether genome-wide significant loci of type 2 diabetes mellitus are associated with the risk of AD”	“The results suggest that genome-wide significant loci of type diabetes (insulin resistance) play no major role in the risk and cognitive impairment of AD”	Low-moderate
Schrijvers et al.^21^	2010	Cohort	3,139	“Investigate whether fasting glucose and insulin levels and IR are associated with the risk of AD and whether this risk is constant over time”	“The study suggests that insulin metabolism influences the clinical manifestation of AD only within 3 years”	Good-quality
Willette et al.^22^	2015	Cross-sectional	186	“Assess whether the IR is associated with amyloid binding in three AD-sensitive brain areas”	“The study demonstrated that IR may contribute to amyloid deposition in brain regions affected by AD”	Good-quality
Morris et al.^23^	2014	Cross-sectional	42	“To compare IR in aging and aging-related neurodegenerative diseases, and to determine the relationship between IR and gray matter volume in each cohort using an unbiased, voxel-based approach”	“The study supports a potential relationship between IR and brain structure in both normal aging and diagnosed neurodegenerative disease”	Good-quality
Kapogiannis et al.^24^	2015	Cross-sectional	26	“To assess brain IR in AD by level of serine-type 1 insulin receptor substrate (IRS-1) and its state of phosphorylation in neural-derived plasma exosomes”	“Insulin resistance reflected in R values from IRS-1 is higher for patients with AD, and accurately predicts development of AD up to 10 year prior to clinical onset”	Good-quality
Willette et al.^25^	2015	Cross-sectional	150	“To determine if IR predicts AD-like global and regional glucose metabolism deficits in late middle-aged participants at risk for AD, and to examine if IR predicts variation in regional glucose metabolism is associated with worse cognitive performance”	“The results show that IR is associated with significantly lower regional cerebral glucose metabolism, which in turn may predict worse memory performance”	Good-quality
Johansson et al.^26^	2013	Cross-sectional	80	“Assess whether the serum but not cerebrospinal fluid levels of insulin-like growth factor-I (IGF-I) and IGF-binding protein-3 are increased in AD”	“Patients with AD as well as other dementias had high levels of IGF-I in serum but not in CSF. In AD patients, the IGF-I system was associated with biomarkers of AD disease status”	Good-quality
Faqih et al.^27^	2021	Cohort	356	“Study the association between AD and IR and the relation between AD and diabetic patients treated with insulin”	“The results suggest that AD is associated with IR”	Good-quality
Hong et al.^28^	2021	Cohort	5,586,048	“Study the potential relationships between the triglyceride glucose index and dementia”	“Triglyceride glucose index was associated with an increased risk of dementia, including AD”	Good-quality

Abbreviations: QUADAS, Quality Assessment of Diagnostic Accuracy Studies; AD, Alzheimer’s disease; IR, insulin resistance; DM2, type 2 diabetes mellitus; IGF, insulin-like growth factor; IRS, insulin receptor substrate.

### Bias and quality of included studies

Concerning bias, we evaluated the included studies utilizing the National Institutes of Health’s quality of studies evaluation tools for cross-sectional, observational cohort, and case-control studies. Of the 17 articles, 14 were regarded as of good quality, while the remaining three were rated as having low-moderate quality.

### Association between AD and BIR

Out of the evaluated studies, one case-control^20^ and one cohort study^19^ concluded that there is no association between AD and BIR.

## DISCUSSION

The connection between AD and BIR is shown by the 17 studies in this systematic review. There is growing evidence indicating that variability in insulin levels and IR are likely to play a substantial function in both the clinical symptoms and pathophysiology of AD^28^ ^,^ ^29^. It is suggested that insulin affects memory, which is regulated by the hippocampus and the adjacent medial temporal cortex^30^ ^,^ ^31^ ^,^ ^32^. AD is the most prevalent type of dementia that occurs in older adults and is determined by notable neuropathological and cognitive impairments. In addition, AD patients are more susceptible to modifications in insulin physiology than those without the disease, which may increase their risk of developing IR and hyperinsulinemia^33^ ^,^ ^34^. The 17 papers selected in this systematic review examined the relationship between IR and cognitive decline in AD. The studies explored various aspects, including alterations in brain networks, genetic associations, metabolic changes, and the impact of IR on glucose metabolism and gray matter volume. They also investigated the role of insulin receptor substrate dysfunction and insulin-like growth factor levels in AD. Additionally, the risk of developing AD in individuals with DM2 and the association between IR and dementia were explored. Overall, the papers highlighted the detrimental effects of IR on cognition and emphasized the potential therapeutic implications of targeting insulin activity in AD. Out of the evaluated studies, one case-control^20^ and one cohort study^19^ concluded that there is no association between AD and BIR.

The association between AD and IR may be affected by various patient-related factors, such as additional clinical data and age. Multiple researchers have explored the proposed associations, and their conclusions align with the current revision, providing evidence that the frequency of AD is higher in obese individuals and DM2 patients. Mechanisms that are known to emerge in these disorders are also becoming apparent ^35^ ^,^ ^36^.

Several researchers reported an increase in burden from DM2, non-alcoholic steatohepatitis, obesity, and AD in recent years^37^. Patients with metabolic syndrome have an increased hazard of dementia, cognitive impairment, or AD^38^. AD is related to progressive BIR and insulin deficiency. The use of intranasal insulin or insulin sensitizer agents has resulted in the recovery of cognitive activity in experimental models, and cognitive disturbance in human AD cases^39^. DM2 and AD share several molecular, biochemical, and mechanical abnormalities^40^. A majority of case-control studies in this research identified a significantly higher presence of metabolic syndrome in AD patients, who also have abnormalities in their glucose metabolism when compared to those without AD. However, in our study, only the abnormal glucose metabolism of metabolic syndrome was statistically related to AD, except for the papers of Abner et al.^19^ and Chung et al.^20^.

Insulin, a peptide hormone primarily secreted by pancreatic β cells, is widely recognized for its pivotal role in regulating glucose metabolism outside the central nervous system (CNS)^41^. However, its significance extends beyond peripheral tissues as it assumes a multifaceted function within the CNS^42^. Under normal physiological conditions, insulin can efficiently traverse the blood-brain barrier (BBB) through a receptor-mediated transport mechanism, with transport rates influenced by various factors like obesity and inflammation. Furthermore, certain brain regions, such as the hypothalamus, that lack BBB protection, can also be accessed by insulin^43^. The production of insulin within the CNS remains a subject of ongoing debate, although rodent studies have observed the presence of insulin mRNA in the brain, as well as the release of insulin from GABAergic interneurons and choroid plexus epithelial cells.

Insulin resistance can be characterized as the insufficient reaction of target tissues to insulin stimulation^44^. The assessment of IR in peripheral tissues is commonly done through the HOMA-IR^45^. For a more accurate evaluation, the hyperinsulinemic-euglycemic clamp (HI-EG) technique is considered the gold standard^46^. This involves a continuous intravenous infusion of insulin at a constant rate, while glucose infusion is adjusted to maintain euglycemia. Greater rates of glucose infusion indicate higher levels of insulin sensitivity. To investigate the effects of insulin on the brain and the connection between brain insulin sensitivity and peripheral insulin sensitivity, the HI-EG clamp technique has been combined with various imaging techniques such as magnetic resonance imaging, electroencephalography, and magnetoencephalography. However, it should be noted that one limitation of the HI-EG clamp is the potential reduction of insulin transport across the BBB, which may occur due to chronic hyperinsulinemia or other factors associated with peripheral IR. As a result, there remains some uncertainty regarding the amount of insulin that reaches the CNS during the clamp procedure. More recently the triglyceride-glucose index is an index that associates the results of triglycerides and fasting glucose for IR evaluation^47^. In our study, the HOMA-IR^45^ was the most frequently used index to evaluate IR among the studies assessed. Similarly, the MMSE^48^ was the most commonly employed standard for assessing AD.

Insulin receptors are present in all brain cell types with varying levels of manifestation throughout different regions. The high spread of insulin receptors indicates that insulin signaling plays diverse roles in the brain^49^ ^,^ ^50^. Although glucose is the primary cerebral energetic origin, its capture and use by neurons are exclusively exercised by insulin and do not need it. GLUT3 is the main glucose carrier in neurons, and its offer is associated with local cerebral energy requirements. Insulin is not necessary for GLUT3-mediated glucose loading^51^.

Studies show a connection between DM2 and brain disorders, in particular AD and intellectual deficits. Cognitive impairment in individuals with DM was recognized nearly a century ago, with the first protocol studies being carried out in the 1980s^52^. These studies reported that higher hemoglobin A1c levels were associated with more severe deficiencies, including memory impairments^53^. Posterior studies confirmed the same evidence and described moderate deficiencies in various cognitive functions. The grade of intellectual commitment is directly related to the duration of DM, diabetic complications, poor glycemic control, hypertension, and depression^54^. It is not clear if DM2 intellectual commitment and dementia are exclusively associated with aging, neurological degeneration, or cerebrovascular effects. Development studies conducted in adolescents and young adults carriers of DM2 present brain structural modifications and intellectual changes, suggesting that development studies conducted in adolescents and young adults carriers of DM2 present brain structural modifications and intellectual changes, suggesting that early disease processes play a part in pathogenesis^55^.

Thus, our systematic review was conducted to explore the link between BIR and AD. The review involved an extensive search of electronic databases, which yielded a total of 520 articles. Of these, only 17 works met the inclusion criteria and were selected for analysis. The studies included in the review were heterogeneous in terms of methodology, sample size, and study population. However, they all investigated the association between BIR and AD using various approaches in human clinical studies.

Although the studies provide valuable insights, certain biases need to be taken into consideration. The design of the case-control studies may be prone to recall and selection bias, which could affect the accuracy of the results. Furthermore, the studies did not explore potential mechanisms that could explain the link between IR, DM2, and AD. The cohort studies investigated the association between fasting glucose, insulin levels, IR, and the risk of AD over time, which provides valuable insights into the association between insulin metabolism and AD risk; however, it is essential to recognize the limitations such as those associated with self-reporting bias, limited generalizability, potential measurement bias, unmeasured confounders, and the restricted timeframe of the study. Concerning the cross-sectional studies design, it only allows for the observation of associations at one point in time, which limits the ability to establish causal relationships between IR and AD development. A longitudinal study design would be more appropriate to assess the predictive value of IR for AD. Although the study by Kapogiannis et al.^24^ provides valuable insights into the association between IR and AD development, it is important to acknowledge the biases and limitations inherent in the study design.

Ultimately, based on the results obtained from the evaluation of the included studies using quality assessment tools, it can be concluded that the majority of the studies exhibited good quality. However, there were some studies classified as having low to moderate quality. Notably, conflicting results were observed regarding the association between AD and BIR, with some studies suggesting no association. Therefore, it can be concluded that the association between AD and BIR remains inconclusive, as there is no strong evidence of their association to date, since the studies are heterogeneous, with some inconclusive results and divergent data in the literature.
